# Enteric and Fecal Methane Emissions from Dairy Cows Fed Grass or Corn Silage Diets Supplemented with Rapeseed Oil

**DOI:** 10.3390/ani11051322

**Published:** 2021-05-05

**Authors:** Mohammad Ramin, Juana C. Chagas, Hauke Smidt, Ruth Gomez Exposito, Sophie J. Krizsan

**Affiliations:** 1 Department of Agricultural Research for Northern Sweden, Swedish University of Agricultural Sciences (SLU), Skogsmarksgränd, 90183 Umeå, Sweden; juana.chagas@slu.se (J.C.C.); sophie.krizsan@slu.se (S.J.K.); 2 Laboratory of Microbiology, Wageningen University & Research, 6708 WE Wageningen, The Netherlands; hauke.smidt@wur.nl (H.S.); ruth.gomezexposito@wur.nl (R.G.E.)

**Keywords:** corn silage, dairy cows, fermentation, grass silage, methane emissions, rapeseed oil

## Abstract

**Simple Summary:**

In this study, we evaluated methane emissions from dairy cows fed grass or corn silage diets supplemented with rapeseed oil. Enteric methane emissions decreased on adding rapeseed oil to the diet, but methane emissions from feces of dairy cows fed diets supplemented with rapeseed oil did not differ. Thus, no trade-offs were observed between enteric and fecal methane emissions due to forage type or addition of rapeseed oil to diets fed to Swedish dairy cows.

**Abstract:**

This study evaluated potential trade-offs between enteric methane (CH_4_) emissions and CH_4_ emissions from feces of dairy cows fed grass silage or partial replacement of grass silage with corn silage, both with and without supplementation of rapeseed oil. Measured data for eight dairy cows (two blocks) included in a production trial were analyzed. Dietary treatments were grass silage (GS), GS supplemented with rapeseed oil (GS-RSO), GS plus corn silage (GSCS), and GSCS supplemented with rapeseed oil (GSCS-RSO). Feces samples were collected after each period and incubated for nine weeks to estimate fecal CH_4_ emissions. Including RSO (0.5 kg/d) in the diet decreased dry matter intake (DMI) by 1.75 kg/d. Enteric CH_4_ emissions were reduced by inclusion of RSO in the diet (on average 473 vs. 607 L/d). In 9-week incubations, there was a trend for lower CH_4_ emissions from feces of cows fed diets supplemented with RSO (on average 3.45 L/kg DM) than cows with diets not supplemented with RSO (3.84 L/kg DM). Total CH_4_ emissions (enteric + feces, L/d) were significantly lower for the cows fed diets supplemented with RSO. Total fecal CH_4_ emissions were similar between treatments, indicating no trade-offs between enteric and fecal CH_4_ emissions.

## 1. Introduction

The agriculture sector is a significant contributor to methane (CH_4_) emissions. Considering that CH_4_ emissions are 28-fold more effective in trapping heat than carbon dioxide (CO_2_) emissions, efforts to reduce CH_4_ emissions are urgently needed [1]. The majority of CH_4_ emissions produced by dairy cows are from enteric fermentation, and can result in losses of up to 12% of the animal’s gross energy [2]. However, CH_4_ emissions from dairy cow manure stored in the cool-temperate European climate are estimated to be approximately 12% of total CH_4_ emissions [3]. The contribution of manure to greenhouse gas (GHG) emissions depends on many factors such as manure chemical composition (which depends on the chemical composition of the diet), storage conditions, and temperature [4]. Sweden and some other Nordic countries use the IPCC Tier 2 methodology in estimating CH_4_ emissions from manure [5]. One main factor in predicting (influencing) CH_4_ emissions from manure is the amount of volatile solids in manure. Volatile solids are organic material in the feces of dairy cows, with the actual content determined by the diet fed to the cows. Higher feed intake and lower digestibility increase the volatile solid content in the manure. Higher indigestible neutral detergent fiber (iNDF) in the diet leads to reduced digestibility that also affects the amount of enteric CH_4_ emitted, as shown by Ramin et al. [6] on replacing barley with oats in diets fed to dairy cows.

Having more organic matter (OM) in the manure could be desirable if the manure is used for biogas purposes. In biogas production, fermentable material is converted to CH_4_, which could serve as a renewable energy source [7]. Since enteric CH_4_ emissions are the major contributor to total CH_4_ emissions, efforts have been made to develop and test strategies to reduce enteric CH_4_ emissions from dairy cows [8]. However, the dietary strategy used to reduce enteric CH_4_ emissions may increase CH_4_ emissions from the stored manure. The chemical composition of the diet can also influence the extent of CH_4_ emissions from stored manure [9]. Few studies have examined the effect of diet on CH_4_ emissions from manure while evaluating the inhibitory effects on enteric CH_4_ emissions at the same time [9]. 

The starting hypothesis in this study was that forage replacement (partial replacement of grass silage by corn silage) and supplementation with rapeseed oil (RSO) in the diet of dairy cows will reduce enteric CH_4_ emissions, but that RSO-supplemented diets will lead to more fecal CH_4_ emissions. The aim of the study was to evaluate possible trade-offs between mitigation of enteric and feces CH_4_ emissions from dairy cows fed a grass or grass silage-corn silage diet, with or without rapeseed oil supplementation. 

## 2. Materials and Methods

All animals were registered and cared for according to guidelines approved by the Swedish University of Agricultural Sciences Animal Care and Use Committee and the National Animal Research Authority (Number A17/16). The experiment was carried out in accordance with the laws and regulations controlling experiments performed with live animals in Sweden.

Samples and data were obtained for eight cows (2 blocks) out of a total of 20 cows included in a production trial investigating the effect of partial replacement of grass silage (GS) by corn silage (CS), without or with RSO supplementation. Details of animals, silages, experimental procedures, and analyses performed in the production trial can be found in Chagas et al. [10]. Feces from eight cows were collected over the last three days of the sampling week, by grab sampling two times/day, exactly during milking, on three consecutive days. At the end of each period, the six feces samples obtained for each cow were pooled.

### 2.1. Experimental Design and Fecal CH_4_ Measurements

Eight red Swedish dairy cows averaging 59.6 ± 39.46 days in milk and 34.7 ± 7.85 kg milk/day pre-trial were blocked (two blocks) by parity and milk yield, and assigned to a 4 × 4 Latin square design with a 2 × 2 factorial arrangement. Dietary treatments were GS, GS supplemented with RSO (0.5 kg/d) (GS-RSO), GS plus CS (GSCS), and GSCS supplemented with RSO (GSCS-RSO), as presented in Table 1. The production study was carried out over four 28-day periods, and the last 10 days of each period were used for data collection and determination of dry matter intake (DMI), milk production, and enteric CH_4_ emissions.

**Figure 1:**
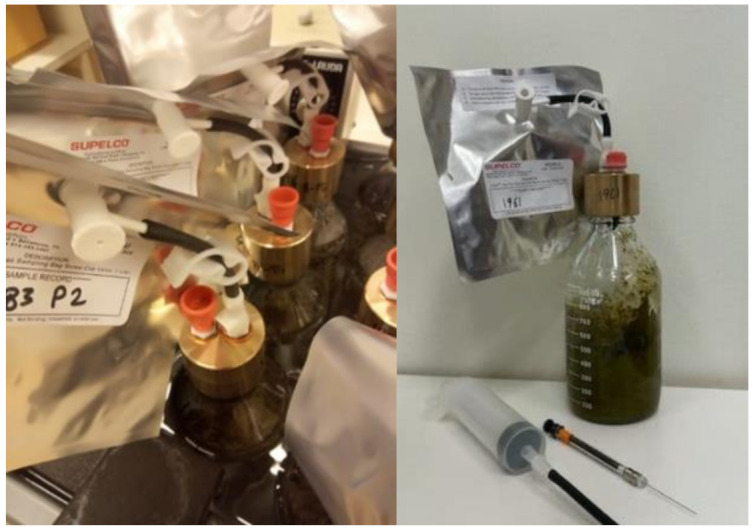
Modified in vitro system for determination of long term methane emissions from feces (nine weeks incubation).

To estimate CH_4_ emissions from feces after nine weeks of incubation, a modified version of the in vitro method described by Ramin and Huhtanen [11] was used. In the modified version, one liter bottles were used and that total gas (cumulative) was recorded manually with a syringe. A small portion (15 g) of the feces was also stored and kept at −80 °C for later microbial analysis. Fresh feces (400 g) from each cow (pooled sample) were placed in 1-L serum bottles connected to gastight bags (SupelTM-Supelco) and incubated at 39 °C for approximately nine weeks (Figure 1). We used 39 °C since it is a standard temperature used for in vitro studies and to speed up the fermentation procedure to estimate potential CH_4_ emissions from feces. Incubations were carried out at the end of each period (1 to 4). The GreenFeed method was used to measure enteric CH_4_ emissions (GreenFeed system, C-Lock Inc., Rapid City, SD, USA), as described by Huhtanen et al. [12]. Methane from feces was measured by sampling the gas in the gastight bags on days 1, 4, and 7 and then every week over the nine weeks. Cumulative CH_4_ emissions were then computed. Methane emissions at each time point were calculated as:

Methane (mL): HSCH_4_ × 600 + Bag CH_4_ × Total gas produced (mL) (1)

where HSCH_4_ is the headspace CH_4_ concentration; Bag CH_4_ is the CH_4_ concentration in the sampling bag; and 600 is the headspace volume in the serum bottles.

The concentration of CH_4_ in gas samples was determined by injecting 0.2 mL of gas withdrawn from the headspace/sampling bag into a gas chromatograph using a gastight syringe, as described by Ramin and Huhtanen [11]. At the end of the incubation, samples of the liquid phase were taken and stored at −20 °C for later determination of volatile fatty acids (VFA). Individual VFA concentration in samples was determined using a Waters Alliance 2795 UPLC system, as described by Puhakka et al. [13].

### 2.2. Chemical Analysis and Maximum CH_4_ Emission Potential

The DM concentration in feed and feces (before and after nine weeks of incubation) was determined by oven drying at 105 °C for 16 h, followed by ash determination by combustion of the dried samples at 500 °C for 4 h [14]. Feed and feces samples were ground using a cutter mill (SM 300, Retsch Ltd., Haan, Germany) to pass through a 1-mm sieve for chemical composition analysis. For the digestibility determinations, another subsample was ground with a mortar and pestle to pass through a 2-mm sieve for iNDF determination. The NDF concentration for both feed and feces samples was determined using heat-stable α-amylase and sodium sulphite [15] (aNDFom), in an Ankom200 Fiber Analyzer (Ankom Technology Corp., Macedon, NY, USA). Potentially digestible NDF (pdNDF, g/kg DM) was calculated as the difference between NDF and iNDF. Neutral detergent solubles (NDS) were calculated as the difference between OM and NDF. Potentially digestible OM (pdOM) was calculated as NDS pdNDF. Indigestible NDF concentration was determined for feed and feces samples as described by Huhtanen et al. [16]. Triplicate portions of 2 g (2 mm) were weighed into polyester bags of 11 μm pore size [17] and incubated for 288 h in the rumen of three cannulated cows fed a grass silage-based diet (60:40 forage to concentrate ratio). The iNDF was expressed exclusive of residual ash. The iNDF content was analyzed for dried feces before and after nine weeks of incubation. Organic matter digestibility (OMD) using iNDF as a marker was calculated according to Equation (2). The same equation was used for the other parameters.

OMD = 1000 − 1000 × [(iNDF in diet DM (g/kg))/(iNDF in fecal DM (g/kg))] [OM in fecal DM (g/kg)]/[OM in diet DM (g/kg)](2)

Daily fecal DM output was calculated as the ratio of iNDF concentration in feces to daily iNDF intake.

Maximum CH_4_ emissions potential based on VFA stoichiometry was calculated using the VFA content determined for feces samples at the end of the 9-week incubations and the buswel CH_4_ potential coefficients taken from Lima et al. [18]. Apparent OMD was calculated as OM fermented/OM in feces before the nine weeks of incubation.

### 2.3. In Vitro Gas Production Measurements

In order to support the results from the long-term incubation of feces (9-week), samples of dried feces from eight cows collected from each period were also used in an in vitro gas production experiment. For this, 1 g of dried feces (1 mm screen) was used as the substrate and three in vitro runs were conducted as described by Ramin and Huhtanen [11]. The in vitro experiment lasted for 72 h, and CH_4_ and total gas were measured at 72 h.

### 2.4. Microbial Analysis

#### 2.4.1. DNA Isolation

Feces (0.12 g of ground material, before nine weeks of incubation) were transferred to a screw-cap tube containing 0.25 g of 0.1 mm glass beads and three 2.5 mm glass beads, and 700 µL of stool transport and recovery (STAR) buffer (Roche Diagnostics) were added. The samples were then subjected to repeated bead beating (5.5 m/s, 3 × 60 s), followed by 15 min heating at 95 °C and 1000 rpm, and centrifuged for 5 min at 4 °C at 21,000 × g. The supernatant was transferred to a separate tube and kept cold. The pellet was subjected to a second round of cell lysis with another 300 µL of STAR buffer. Supernatants from both cycles were pooled, and 250 µL were eluted in 50 µL of nuclease-free water and used for DNA isolation with a Maxwell 16 Tissue LEV Total RNA Purification Kit (Promega, Madison, WI, USA). Negative controls using only reagents and no sample were also included. The quantity of DNA was fluorometrically determined using Qubit in combination with the dsDNA BR Assay Kit (Invitrogen, Carlsbad, CA, USA) following the manufacturer’s recommendations. The DNA obtained was diluted to ~20 ng/µL and stored at −20 °C until further use.

#### 2.4.2. 16S rRNA Gene Amplicon Sequencing

Microbiota composition was analyzed with barcoded amplicons of the V4 region of the 16S rRNA gene generated using the F515–806R primer set [19]. The amplification reactions were performed in triplicate as described elsewhere [20]. After confirmation of amplicon size by agarose gel electrophoresis, PCR products were purified with HighPrep (MagBioEurope Ltd, Kent, UK) following the manufacturer’s instructions. PCR products were pooled in equimolar amounts and sequenced on the Illumina NovaSeq 6000 platform (GATC-Biotech, Konstanz, Germany). To control for potential technical biases, two human gut mock synthetic communities [21] and three rumen mock synthetic communities (manuscript in preparation) were included as positive controls, and PCR reactions with no DNA template as negative controls. 

#### 2.4.3. Microbiota Composition Analysis

Raw sequences were processed in the NG-Tax 2.0 pipeline [22], using the default settings and the SILVA 132 SSU reference database [21]. Read counts were normalized to relative abundance using the R software (version 3.5.0). The data were then subjected to statistical analysis using the SAS program. The raw sequence data generated for this study can be found in the European Nucleotide Archive (ENA) under accession number PRJEB43237.

### 2.5. Statistical Analysis

The data were analyzed using the GLM procedure in SAS (release 9.2; SAS Institute Inc., Cary, NC, USA) by applying the following model:

Yijkl = μ + Ci + Pj + Bk + Dl + eijkl(3)

where Yijkl is the dependent variable; µ is the overall mean; Ci is the effect of cow I; Pj is the effect of period j; Bk is the effect of block k; Dl is the effect of diet l; and eijkl ~N(0,$\sigma_{e}^{2}$) is the random residual error. The same model was used for in vitro gas production of CH_4_ and total gas from dried feces, considering only diet in the model and including run as a random variable.

For diet comparisons, the following orthogonal contrasts were used: the effect of forage source (GS or CS), the effect of RSO supplementation, and the interaction of forage × oil supplementation. Differences were considered significant at *p* ≤ 0.05.

## 3. Results

### 3.1. Chemical Composition of Feces and Production Data

Chemical composition of the diets used is shown in Table 1. The grass silage diet supplemented with RSO tended to have a lower iNDF value than the other diets, while pdNDF content was slightly higher for the GS diet. The GS-RSO and GSCS-RSO diets showed greater amounts of crude fat compared to other diets not supplemented with RSO (Table 1). The NDF (g/kg DM) concentration in feces was 475, 476, 515 and 497 for GS, GS-RSO, GSCS and GSCS-RSO, respectively. Including RSO in the diet decreased DM intake by 1.75 kg/d (*p* < 0.01) and partial replacement of GS by CS showed a tendency to reduce DMI by 1.2 kg/d (Table 2). A similar decline was observed for nutrient intake (Table 2). Milk yield declined by 1.9 kg/d with partial replacement of GS by CS (Table 2). There was no effect on fecal DM output from inclusion of RSO in the diet (Table 3). Potential digestible OM was greater with the inclusion of RSO in the diet (on average 3.83 vs. 3.35 kg/d) (Table 3).

### 3.2. Fermentation and Digestibility

Total VFA production was similar at the end of fermentation (nine weeks), indicating that there were also no differences between the treatments in the second stage of CH_4_ emissions potential (Table 3). Digestibility estimated using iNDF before nine weeks of incubation indicated differences caused by RSO supplementation and forage type for DMD, OMD, NDFD, and pdNDFD (Table 3). Supplementation of RSO and partial replacement of GS by CS reduced DM, OM, NDF, and pdNDF digestibility (Table 3).

The effect of reduced digestibility for the RSO supplementation diets persisted only for DMD and OMD when iNDF after nine weeks of incubation was used for the calculations (Table 3). Apparent OMD did not differ between treatments (Table 3).

### 3.3. Methane Emissions

**Figure 2:**
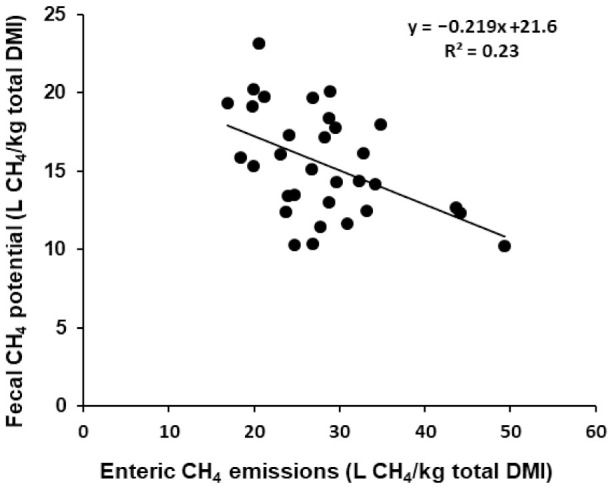
Relationship between enteric CH_4_ emissions and maximum CH_4_ emissions potential from feces (*n* = 32). Fecal CH_4_ potential was predicted from VFA (mM) stoichiometry, based on VFA determined for the samples and the buswel CH_4_ potential coefficients taken from Lima et al. [18].

Enteric CH_4_ emissions were reduced by inclusion of RSO in the diet (on average 473 vs. 607 L/d; Table 3). In in vitro incubation of feces for nine weeks, there was a trend for lower CH_4_ emissions from feces of cows with RSO-supplemented diets (on average 3.45 vs. 3.84 L/kg DM; Table 3), indicating no trade-offs between enteric and feces CH_4_ emissions due to the dietary strategies tested. However, CH_4_ emissions (L/kg OM) were significantly lower (*p* < 0.02) from the feces of cows with RSO-supplemented diets (Table 3). Total feces CH_4_ emissions (L/d) did not differ between the treatments (Table 3). Total emissions of CH_4_ (enteric + feces) were significantly lower (*p* < 0.01) with dietary supplementation with RSO (Table 3). There was a weak relationship between enteric CH_4_ emissions and maximum CH_4_ emissions potential calculated from VFA in relation to DMI (Figure 2).

The results of the in vitro gas production of dried feces (before nine weeks of incubation) showed no differences between diets in potential CH_4_ emissions (L/kg DM) at 72 h of incubation (Table 4). The effect was also not significant for total gas production and ratio of CH_4_ emission/total gas (Table 4). The ratio of archaeal over bacterial 16S rRNA gene sequences as a measure of the relative contribution of both phyla to the fecal microbiota did not differ between cows fed the different diets (Table 5). In analyses of microbial composition of feces before nine weeks of incubation, the relative abundance of Archaea did not show significant differences related to RSO supplementation. Interestingly, however, Methanocorpusculum was only detected when cows were fed the GS-RSO diet. The relative abundance of some of the 15 most abundant bacterial families was significantly affected by forage type and RSO supplementation (Table 5).

## 4. Discussion

In this study, we evaluated CH_4_ emissions from the feces of dairy cows fed GS only or GS partly replaced by CS, in both cases with/without supplementation with RSO. Dairy cows fed the GSCS diet with RSO were expected to have higher CH_4_ emissions from feces, due to deposition of more fermentable substrates to the feces since inclusion of oil will reduce the digestibility. Since crude fat concentration was greater in the diets supplemented with RSO, the results confirmed that inclusion of RSO in the diet significantly reduced enteric CH_4_ emissions and digestibility. It is well documented that dietary fat concentration has a negative relationship with enteric CH_4_ emissions and consequently on digestibility. Dietary fat can influence enteric CH_4_ emissions through different mechanisms. Increasing fat concentration in the diet replaces fermentable substrates with non-fermentable substrates in the diet. It also affects the fiber digestibility and the biohydrogenation of dietary unsaturated fatty acids acts as a H_2_ sink in the rumen.

However, total CH_4_ emissions (L/d) from feces did not differ between the dietary treatments tested, indicating no trade-offs between enteric and feces CH_4_ emissions.

### 4.1. Chemical Composition of Feces and Digestibility

Partial replacement of GS by CS resulted in a numerical increase in OM output in the feces. Similarly, Benchaar and Hassanat [23] found that dairy cows fed midrib corn silage excreted more volatile solids than cows fed conventional corn silage. The OM output in the present study was also numerically higher for the diets supplemented with RSO. Partial replacement of GS by CS increased NDF excretion in feces, as also observed by Hassanat and Benchaar [4] for cows fed a CS diet compared with a red clover silage diet. Similarly, Massé et al. [24] found that replacement of alfalfa silage by corn silage in the diet of dairy cows increased the NDF output in the feces.

Cows fed the GSCS-RSO diet tended to show greater OM output in the feces (5.2 kg/d) than cows fed the other three diets. This was close to the value suggested by IPCC [5] of 5.4 ± 1.1 kg/d. The NDF digestibility using iNDF from feces before nine weeks of incubation was lower for the GSCS diet than for the GS diet, as also observed by Benchaar et al. [25] as a result of higher starch with diets including CS. Reduced NDF digestion as a result of feeding CS-based diets has also been reported by Brask et al. [26] and Arndt et al. [27] in studies where cows were fed a high-starch diet (CS-based) compared with GS or legume-based diets. However, other studies have found that supplementation of oil in grass silage-based diets has no effect on digestibility [28]. This is in line with the findings in the present study when iNDF for feces after nine weeks of incubation was used for digestibility determinations. Benchaar et al. [25] showed that supplementation of linseed oil to cereal-based diets for dairy cows lowered the digestibility of nutrients, but no effect on digestibility was observed when using forage or legume-based diets.

Diets supplemented with RSO (GS-RSO and GSCS-RSO) showed lower iNDF output in the feces compared with diets not supplemented with RSO (on average 1.11 vs. 1.21 g/kg DM). Since iNDF is not digestible, the decline in digestibility with RSO supplementation in the diets is in line with higher amounts of pdNDF and pdOM in the feces output, as shown in Table 3. Benchaar et al. [25] reported decreased content of lignin in the feces of cows fed a CS-based diet. However, in the present study, partial replacement of GS by CS numerically increased the iNDF content in the feces. One reason why Benchaar et al. [25] observed decreased content of lignin in feces samples from cows fed a CS diet could be that they used a CS diet, while in the present study, GS was only partly replaced by CS.

### 4.2. Fermentation in Incubated Feces

The pH at the end of incubation was higher for the GS diet compared with the GSCS diet. In contrast, Hassanat and Benchaar [4] observed no differences in pH when comparing a CS diet with a red clover silage diet. The total VFA concentration was not different between the treatments in the present study, but Hassanat and Benchaar [4] reported greater total VFA concentrations in the manure of cows fed a CS diet in the first week of incubation. However, the effect was not different for the remaining weeks of incubation. We also found no effect on total VFA concentration between the treatments at the end of incubation, except that acetate concentration remained lower for the GSCS diet compared with the GS diet. Hassanat and Benchaar [4] reported higher acetate values for their CS diet during the first week of incubation. The increase in acetate and total VFA has been attributed to the activities of acetogenic bacteria in degrading OM to VFA [29,30]. In a study by Ramin et al [31], fecal inoculum showed lower hydrogen recoveries compared with rumen inoculum, suggesting the existence of acetogenesis in the hindgut. Lu et al. [32] found a direct relationship between total VFAs and CH_4_ emissions. This is in line with our findings, since the effect of forage and oil supplementation was not significant between different treatments when comparing total VFAs and total feces CH_4_ emissions (L/d). Moreover, reductions in CH_4_ emissions have often been associated with increments in propionate concentration and reductions in acetate concentration [33]. Similarly, in the present study, the effect of RSO supplementation was not significant for molar proportions of VFAs, as also found for the effect on total feces CH_4_ emissions (L/d).

### 4.3. Methane Emissions from Feces

The results from the gas in vitro system (72 h incubation, Table 4) showed no differences in CH_4_ potential from the feces of cows fed the different diets, regardless of forage type or RSO supplementation, confirming findings in the 9-week incubations of fresh feces in closed fermentation units. The conventional in vitro system used in this study has been proven to give reliable estimates of CH_4_ emissions, comparable to those in in vivo studies using a respiration chamber to measure CH_4_ emissions [34].

Studies examining the archaeal and bacterial community in cow feces fed CS or a diet supplemented with RSO are rare. In a study by Mu et al. [35] where rumen and fecal communities in high- and low-yielding dairy cows were examined, differences were observed in the ruminal bacteria, but not in the bacteria from feces. However, the animal-specific microbes existing in the rumen were within a minor group, and between-animal variation is more pronounced in fecal communities [36]. In the present study, microbiota data from the fecal community were used to relate data obtained from fecal incubation (here CH_4_ emissions) to differences in the relative abundance of archaeal and bacterial community members in feces before nine weeks of incubation. Most studies have reported a negative relationship between fat and enteric CH_4_ emissions [37,38].

In line with the CH_4_ data, the microbial data (Table 5) showed no significant differences in the abundance of archaea in the feces of cows fed different diets. Recently, Bayat et al. [28] showed reductions in the abundance of archaea in the rumen when cows were fed a GS diet supplemented with RSO compared with a control diet with no RSO supplementation. Similarly, cows fed the GS-RSO and GSCS-RSO diets showed reductions in enteric CH_4_ emissions of around 22% compared with cows fed the GS and GSCS diet (134.5 L/d less on average). In the present study, CH_4_ emissions (L/kg OM) from feces showed a similar trend, whereas Hassanat and Benchaar [4] recorded greater emissions of CH_4_ from the manure of cows fed a CS diet supplemented with linseed oil. However, in the present study, the feces were incubated for nine weeks (we saw few changes in total gas and CH_4_ emissions from weeks seven to nine) and not mixed with urine or water. The study by Hassanat and Benchaar [4] reported that over up to 12 weeks of manure incubation, CH_4_ emissions from manure from cows fed a red clover silage diet supplemented with linseed oil did not increase compared with emissions from the manure from cows fed a non-supplemented red clover silage diet. However, the manure started to produce more CH_4_ starting from week 13 to the end for the red clover silage diet supplemented with linseed oil [4]. In the present study, maximum CH_4_ emissions potential was predicted based on buswel CH_4_ potential coefficients taken from Lima et al. [18]. The average for all diets was 64.7 L/kg OM feces, which was around 43% lower than the 149 L/kg OM reported by Hassanat and Benchaar [4]. One reason could be that in the present study, VFA samples were taken after nine weeks of incubation, whereas Hassanat and Benchaar [4] used CH_4_ conversion factors suggested by Environment Canada [39]. The IPCC value for maximum CH_4_ emissions potential from manure is much higher than values obtained in many experimental studies [4]. This could be because the IPCC value does not consider that the diet effect and supplementation of the diet with other substances (e.g., oil) could strongly affect the maximum CH_4_ emissions from manure. Møller et al. (2014) reported a negative relationship (R^2^ = 0.57) between enteric CH_4_ emissions and fecal CH_4_ emissions potential when dairy cows were fed two different levels of fat with different roughage types. In the present study, the relationship was weaker (R^2^ = 0.23) and not significant (*p* = 0.08), indicating that addition of RSO to the GS and GSCS diets tended to increase CH_4_ emissions from feces in relation to DMI, but the effect was not as strong as reported by Møller et al. [9].

The difference in total CH_4_ emissions (enteric + feces, L/d) when comparing RSO-supplemented and non-supplemented diets was 120 L/d in this study. This is rather similar to the 95 L/d lower CH_4_ emissions in a study where linseed oil was added to a red clover-CS diet [25]. Taken separately, the enteric and fecal CH_4_ emissions in this study provide evidence that there were no trade-offs between enteric and fecal CH_4_ emissions. However, under normal conditions, many factors can influence CH_4_ emissions from incubated manure [40] including humidity, air temperature, sun, and storage facilities.

## 5. Conclusions

Enteric and fecal CH_4_ emissions from dairy cows fed grass silage or partial replacement of grass silage by corn silage, with/without RSO supplementation, were compared. Total dry matter and nutrient intake were negatively affected by RSO supplementation. Forage replacement did not have any effect on enteric CH_4_ emissions, whereas emissions of enteric CH_4_ were lower for cows fed RSO-supplemented diets. Total CH_4_ emissions from feces (L/d) were not affected by forage replacement and/or RSO supplementation. The abundance of archaea and total CH_4_ emissions from feces were not affected by partial replacement of GS by CS and/or RSO supplementation. No trade-offs were seen between enteric and feces CH_4_ emissions due to the forage replacement and RSO strategy tested in this study.

## Figures and Tables

**Table 1:** Ingredient composition (g/kg of DM) and chemical composition of the experimental diets fed to dairy cows.

Item	Diet ^1^			
	GS	GS-RSO	GSCS	GSCS-RSO
Ingredient				
Grass silage	558	534	277	264
Corn silage	0	0	290	278
Crimped barley	336	316	328	311
Rapeseed meal	91.0	91.0	90.0	90.0
Rapeseed oil	0	44.0	0	42.0
Minerals ^2^	15.0	15.0	15.0	15.0
Chemical composition In dry matter, g/kg				
Organic matter	922	924	935	935
Neutral detergent fiber	371	355	348	332
Indigestible NDF	61.9	59.4	68.8	66.1
pdNDF ^3^	309	296	279	266
Crude fat ^4^	33.8	76.1	33.0	73.1

^1^ Total mixed ration (TMR): GS = grass silage; GS-RSO = grass silage with rapeseed oil; GSCS = grass silage plus corn silage; GSCS-RSO = grass silage plus corn silage with rapeseed oil; In addition to TMR, the cows received a concentrate mixture during visits to the GreenFeed system, crude protein = 194 g/kg DM, Neutral detergent fiber = 248 g/kg DM, and organic matter = 934 g/kg DM; ^2^ Commercial mineral mixture Effekt Optimal (Lantmännen Lantbruk AB, Malmö, Sweden); ^3^ pdNDF = potentially digestible NDF; ^4^ Crude fat was calculated based on previously analyzed fat in grass (Eurofins AB) and maize silage and values reported by the feed company. The ingredients were provided from (Lantmännen Lantbruk AB, Malmö, Sweden).

**Table 2:** Intake and production data for cows fed the experimental diets.

Item	Diet ^1^				SEM	*p*-Value ^3^	
	GS	GS-RSO	GSCS	GSCS-RSO		Forage	Oil
Intake, kg/d							
Dry matter	21.2	19.2	20.0	18.5	0.50	0.07	<0.01
Organic matter	19.5	17.7	18.6	17.2	0.46	0.17	<0.01
Neutral detergent fiber	7.42	6.63	6.64	5.94	0.194	<0.01	<0.01
Indigestible NDF	1.17	1.08	1.26	1.14	0.036	0.06	<0.01
pdNDF ^2^	6.37	5.68	5.45	4.86	0.167	<0.01	<0.01
Milk yield, kg/d	31.5	32.7	29.6	29.2	0.88	<0.01	0.88

^1^ Total mixed ration (TMR): GS = grass silage; GS-RSO = grass silage with rapeseed oil; GSCS = grass silage plus corn silage; GSCS-RSO = grass silage plus corn silage with rapeseed oil; ^2^ pdNDF = potentially digestible NDF; ^3^ Forage × oil interaction was not significant for any parameters *p* ≥ 0.68.

**Table 3:** Fecal output, methane emissions (enteric and from feces), and other fermentation parameters following 9-weeks in vitro incubations of feces from dairy cows fed a grass/corn silage-based diet, with or without rapeseed oil supplementation.

	Diet ^1^					*p*-Value ^10^	
Item	GS	GS-RSO	GSCS	GSCS-RSO	SEM	Forage	Oil
*Fecal output, kg/d*							
Dry matter	5.13	5.35	5.32	5.85	0.232	0.15	0.13
Organic matter	4.45	4.69	4.70	5.22	0.203	0.06	0.07
NDS ^2^	2.01	2.18	1.97	2.32	0.119	0.64	0.04
Neutral detergent fiber	2.44	2.51	2.72	2.89	0.127	0.01	0.33
Indigestible NDF	1.17	1.08	1.26	1.14	0.036	0.06	<0.01
pdNDF ^3^	1.27	1.42	1.46	1.74	0.098	0.01	0.03
pdOM ^4^, kg/d	3.27	3.60	3.43	4.07	0.177	0.09	0.01
*CH_4_ emission*							
Feces, L/kg DM	3.99	3.31	3.70	3.60	0.17	0.98	0.05
Feces, L/kg OM	4.66	3.81	4.22	4.03	0.19	0.60	0.02
Feces, L/d	18.3	16.1	18.6	20.2	1.99	0.30	0.90
Enteric, L/d	608	459	607	487	21.7	0.53	<0.01
Enteric + feces, L/d	637	507	618	508	21.4	0.69	<0.01
Maximum CH_4_ from VFA, L/kg DM feces ^5^	59.4	55.5	58.0	54.4	2.63	0.64	0.16
Maximum CH_4_ from VFA, L/kg OM feces	69.0	63.5	66.0	60.6	2.97	0.32	0.09
Maximum CH_4_ from VFA, L/kg DMI	14.4	15.4	15.2	16.8	0.708	0.14	0.08
*VFA after 9 weeks of in vitro incubation*							
Total VFA, mM	199	196	189	188	6.9	0.22	0.79
Acetate, mmol/mol	608	597	573	575	9.4	<0.01	0.62
Propionate, mmol/mol	191	200	194	198	7.4	0.94	0.40
Butyrate, mmol/mol	172	175	204	200	13.1	0.04	0.96
Isobutyric acid, mmol/mol	27.8	27.0	28.2	27.0	1.29	0.88	0.45
pH after 9 weeks of incubation	5.41	5.18	5.17	5.16	0.049	0.02	0.04
*In vivo digestibility using iNDF from feces before 9 weeks in vitro incubation*							
DMD ^8^, g/ kg	755	719	746	680	7.7	<0.01	<0.01
OMD ^6^, g/ kg	775	741	765	699	6.7	<0.01	<0.01
NDFD ^7^, g/kg	627	575	580	488	10.9	<0.01	<0.01
pdNDFD, g/kg	760	686	719	582	15.5	<0.01	<0.01
*In vivo digestibility using iNDF from feces after 9 weeks in vitro incubation*							
DMD, g/ kg	784	746	770	759	9.3	0.97	0.01
OMD, g/ kg	805	769	790	777	8.4	0.67	<0.01
NDFD, g/kg	669	611	614	610	18.2	0.13	0.11
pdNDFD, g/kg	822	740	767	761	25.8	0.51	0.10
Apparent OMD ^9^, g/kg	213	209	205	201	8.8	0.36	0.59

^1^ GS: grass silage; GSCS = grass silage + corn silage; GSCS-RSO = grass silage + corn silage + rapeseed oil; GS-RSO = grass silage + rapeseed oil; ^2^ Neutral detergent solubles (OM–NDF); ^3^ Potentially digestible NDF calculated as NDF–iNDF; ^4^ Potential digestible organic matter calculated as NDS + pdNDF; ^5^ Predicted from VFA (mM) stoichiometry according to Lima et al. [18]; ^6^ Organic matter digestibility; ^7^ Neutral detergent fiber digestibility; ^8^ Dry matter digestibility; ^9^ Calculated as OM fermented/OM in feces before 9 weeks of storage; ^10^ Forage × oil interaction was not significant for any parameters *p* ≥ 0.36.

**Table 4:** Methane and total gas emissions following incubation in a traditional in vitro gas production system for 72 h of dried feces obtained from dairy cows fed a grass/corn silage-based diet, with or without rapeseed oil supplementation.

Item	Diet ^1^				SEM	*p*-Value ^2^	
	GS	GS-RSO	GSCS	GSCS-RSO		Forage	Oil
Methane emissions, L/kg DM	11.0	12.5	13.4	12.4	1.11	0.31	0.77
Total gas production, L/kg DM	95.0	103	97.0	104	6.08	0.77	0.18
Methane/total gas production	0.118	0.163	0.144	0.122	0.0257	0.78	0.66

^1^ GS = grass silage; GSCS = grass silage + corn silage; GSCS-RSO = grass silage + corn silage + rapeseed oil; GS-RSO = grass silage + rapeseed oil; ^2^ Forage × oil interaction was not significant for any parameters *p* ≥ 0.19.

**Table 5:** Relative abundance of Archaea and the 15 most abundant bacterial families and genera before nine weeks of incubation in feces from dairy cows fed a grass/corn silage-based diet with or without rapeseed oil supplementation.

Abundance (%)	Diet ^1^				SEM	*p*-Value ^2^	
	GS	GS-RSO	GSCS	GSCS-RSO		Forage	Oil
Archaea/Bacteria	4.42	5.26	4.23	4.23	0.781	0.44	0.59
Archaea							
*Methanobrevibacter*	97.5	97.7	98.9	98.5	0.72	0.13	0.92
*Methanosphaera*	2.52	2.16	1.10	1.46	0.722	0.15	1.0
*Methanocorpusculum*	0.0	0.13	0.00	0.00	0.068	0.33	0.33
Top 15 bacterial families							
*Atopobiaceae*	1.44	2.45	2.56	2.71	0.620	0.28	0.36
*Bacteroidaceae*	1.59	1.58	1.46	2.27	0.365	0.45	0.28
*Bacteroidales_RF16_group*	0.93	0.57	1.03	0.87	0.219	0.37	0.24
*Bifidobacteriaceae*	3.04	1.97	8.9	0.72	1.544	0.15	<0.01
*Christensenellaceae*	4.89	5.61	4.93	5.05	0.415	0.53	0.32
*Eggerthellaceae*	1.30	1.65	0.95	0.72	0.239	0.01	0.80
*Erysipelotrichaceae*	2.97	2.88	2.27	2.62	0.369	0.21	0.73
*Family_XIII*	4.29	4.87	3.15	4.01	0.527	0.07	0.18
*Lachnospiraceae*	20.4	24.8	16.2	22.9	1.76	0.10	<0.01
*Lactobacillaceae*	1.48	1.55	0.68	0.70	0.210	<0.01	0.84
*Muribaculaceae*	1.65	2.84	2.30	3.98	0.474	0.07	<0.01
*Peptostreptococcaceae*	6.26	6.47	5.97	5.78	0.744	0.51	0.98
*Prevotellaceae*	9.93	6.45	10.2	10.7	1.10	0.05	0.19
*Rikenellaceae*	8.00	4.54	7.66	5.40	0.709	0.71	<0.01
*Ruminococcaceae*	28.3	26.8	28.1	27.0	1.31	0.96	0.32
Top 15 Bacterial genera							
*Bifidobacterium*	3.04	1.96	8.89	0.72	1.542	0.15	<0.01
*Christensenellaceae_R-7_group*	4.89	5.61	4.93	5.05	0.415	0.53	0.32
*Eubacterium_coprostanoligenes_group*	5.36	7.82	5.77	7.54	0.447	0.88	<0.01
*Lachnospiraceae_NK3A20_group*	12.1	16.2	9.73	10.2	1.27	<0.01	0.09
*Mogibacterium*	2.27	2.48	1.74	2.23	0.237	0.11	0.15
*Olsenella*	1.24	2.15	2.41	2.68	0.614	0.18	0.34
*Paeniclostridium*	2.00	2.08	2.16	1.96	0.292	0.94	0.82
*Prevotellaceae_UCG-003*	5.80	3.77	5.48	6.89	0.864	0.12	0.72
*Rikenellaceae_RC9_gut_group*	6.31	3.72	6.42	4.45	0.686	0.54	<0.01
*Romboutsia*	3.46	3.62	3.08	3.19	0.447	0.38	0.77
*Ruminococcaceae_UCG-005*	14.8	12.5	13.2	10.1	1.03	0.06	0.01
*Ruminococcaceae_UCG-013*	2.23	1.57	3.42	3.34	0.476	<0.01	0.44
*Ruminococcus_2*	1.95	1.12	2.24	2.38	0.764	0.32	0.65
*Turicibacter*	2.12	1.90	1.71	1.76	0.306	0.38	0.79
*Unknown_Lachnospiraceae*	2.17	2.30	1.52	3.19	0.373	0.74	0.02

^1^ GS = grass silage; GSCS = grass silage + corn silage; GSCS-RSO = grass silage + corn silage + rapeseed oil; GSRSO = grass silage + rapeseed oil; ^2^ Forage × oil interaction was not significant for any parameters *p* ≥ 0.59.

## Data Availability

The raw sequence data for the microbial analysis that was generated for this study can be found in the European Nucleotide Archive (ENA) under accession number PRJEB43237.
